# Retrospective Study of Factors Potentially Influencing Occurrence of Cough in Slovak Patients with Sarcoidosis

**DOI:** 10.1155/2019/3808206

**Published:** 2019-06-02

**Authors:** Eva Kovacova, Robert Vysehradsky, Ivan Kocan, Jana Plevkova, Tomas Buday

**Affiliations:** ^1^Clinic of Pneumology and Phthisiology, Martin University Hospital and Jessenius Faculty of Medicine in Martin, Comenius University in Bratislava, Martin, Slovakia; ^2^Department of Pathophysiology, Jessenius Faculty of Medicine in Martin, Comenius University in Bratislava, Martin, Slovakia

## Abstract

**Introduction:**

Sarcoidosis is a multisystem granulomatous disease of unknown aetiology, commonly involving the lungs.  Cough is a frequent and troublesome symptom of sarcoidosis that reduces patients' quality of life.

**Aim:**

Retrospective analysis of different factors—smoking history, Scadding stage, results of lung function testing, calcium metabolism, endobronchial finding, CD4+/CD8+ T-cell ratio in bronchoalveolar lavage fluid (BALF), and other sarcoidosis symptoms in relationship to presence/absence of cough in sarcoidosis patients.

**Methods:**

We retrospectively studied sarcoidosis patients diagnosed at the Clinic of Pneumology and Phthisiology of Martin University Hospital between 1998 and 2018. Patients with a history of cough-relevant comorbidities were excluded from the study. GraphPad Prism 7.0 software was used to perform statistical analysis.

**Results:**

101 sarcoidosis patients were included to the study: 65 patients reporting from cough and 36 without cough. The cough was slightly more frequent in nonsmokers (*p*=0.166) and in women (*p*=0.688). Cough was associated with dyspnoea (*p*=0.0007), fever (*p*=0.0324), and chest pain (*p*=0.0206) and did not associate with arthralgia (*p*=0.317) and erythema nodosum (*p*=0.505). Patients with cough had significantly a lower average value of calciuria (*p*=0.0014) and lower MEF25 (*p*=0.0304), MEF50 (*p*=0.0061), FEV1 (*p*=0.0025), and FVC (*p*=0.0025) in % of predicted values, and more often positive endobronchial finding (*p*=0.0206), compared to patients without cough. Calcemia, FEV1/FVC, DLCO, and CD4+/CD8+ T-cell ratio in BALF and occurrence of cough did not differ between different stages of the disease.

**Conclusions:**

We found significant differences between sarcoidosis patients with/without cough regarding symptoms, results of lung function tests, endobronchial finding, and calcium metabolism. Further research is needed to understand the etiopathogenesis of cough in sarcoidosis patients.

## 1. Introduction

Sarcoidosis is a multisystem disease of unknown aetiology characterized histologically by the presence of noncaseating granulomatous inflammation. Although any tissue of the human body can be affected, sarcoidosis typically affects hilar and mediastinal lymph nodes, the lung interstitium, and bronchial epithelium.

Diagnosis of sarcoidosis relies on a compatible clinical and radiologic presentation (Scadding stage), the histological evidence of noncaseating granulomatous inflammation and exclusion of other diseases with similar findings, such as infections or malignancies. Diagnostic algorithm of pulmonary sarcoidosis includes flexible fibrobronchoscopy with evaluation of macroscopic changes of tracheobronchial tree (presence of inflammation and/or granulomas). Different kinds of endoscopic biopsies can be taken for histologic confirmation of the presence of nonnecrotizing granulomas. Analysis of the cellular component of bronchoalveolar lavage fluid (BALF) is included frequently in the diagnostic algorithm. The proportion of T-cells in BALF greater than 15% has a sensitivity of 90% for the diagnosis of sarcoidosis although the specificity is low [1]. The high CD4+/CD8+ ratio (>3.5) is considered to be highly specific (93–96%) for sarcoidosis and, when found in association with a typical clinical manifestation, may support the diagnosis and obviate the need for confirmation by additional procedures [2, 3]. However, at least 5% of sarcoidosis cases shows low CD4+/CD8+ ratio [4, 5].

Sarcoidosis may encompass numerous different clinical presentations. Symptoms of sarcoidosis typically include increased body temperature/fever, night sweats, fatigue, lymph nodes enlargement, and respiratory symptoms such as cough, dyspnoea, chest pain, wheeze or abnormal breathing phenomena, less frequently haemoptysis, nasal congestion or crusting, epistaxis, and hoarseness. Extrathoracic manifestation includes gastrointestinal symptoms, commonly ocular symptoms, skin lesions (erythema nodosum and many others), cardiac, musculoskeletal, and neurologic symptoms, and symptoms of D vitamin dysregulation [6].

Chronic cough is a troublesome symptom of sarcoidosis, with a negative impact on the quality of life of sarcoidosis patients. Objective 24 h cough monitoring proved that patients with sarcoidosis have significantly higher cough frequency when comparing to healthy controls, and their cough shows diurnal variation pattern, being significantly more frequent during the day compared with the night [7]. The prevalence of cough in patients with sarcoidosis has been estimated to be between 3 and 53%: Japan 3%, Finland 33%, Saudi Arabia 40%, United Kingdom 50%, and Turkey 53% [8]. In a recent study [9], intrathoracic involvement was present in 97% of cases of sarcoidosis. In 87% of cases, this consisted of intrathoracic lymphadenopathy and 50% had evidence of pulmonary parenchymal infiltration, but only 43% had respiratory symptoms.

Little is known about mechanisms of cough in sarcoidosis patients. Consequences of granulomatous inflammation on airway sensory nerves together with the airway obstruction and hyperresponsiveness, concentration, function, and polymorphisms of ACE, and tissue remodelling, bronchiectasis, and fibrosis are discussed as potential cough triggers [10].

In sarcoidosis, activated alveolar macrophages show increased activity of the 25-hydroxyvitamin D 1-alpha-hydroxylase (1-alpha hydrolase) enzyme, which converts vitamin D-25 to vitamin D-1,25 (calcitriol). The great majority of sarcoidosis patients have normal or elevated serum levels of 1,25-dihydroxy vitamin D (calcitriol), that is considered to be a marker associated with disease activity. High calcitriol levels are often associated with hypercalcemia and hypercalciuria. Hypercalcemia has been reported in about 5% of cases of sarcoidosis, but some series have reported hypercalcemia in over a quarter of their cases. Hypercalciuria occurs in up to a third of sarcoidosis patients. Persistent hypercalciuria and/or hypercalcemia can lead to nephrocalcinosis and renal insufficiency. Abnormal calcium metabolism has been reported more frequently in Caucasians than African Americans. It is also more common in men than in women. Abnormal calcium metabolism has also been associated with a chronic disease course [11].

In the recent literature, there is growing evidence about pleiotropic and immunomodulatory effects of vitamin D. It is known that it has an influence on airway smooth muscle function, secretion of inflammatory mediators [12]. Until now, relationships of cough, calcium metabolism, and the level of calcitriol have not been studied so far.

The aim of this retrospective study is to analyse relationship of various factors—gender, smoking history, Scadding stage, results of lung function testing, calcium metabolism, endobronchial finding, CD4+/CD8+ T-cells ratio in bronchoalveolar lavage fluid (BALF), and other common sarcoidosis symptoms (dyspnoea, fever, arthralgia, erythema nodosum, and chest pain)—with the presence/absence of cough in patients with sarcoidosis. Presence of cough in our cohort was obtained during patients' admission to the hospital from their respective medical history.

## 2. Methods

### 2.1. Ethical Approval

This retrospective study was approved by the Ethics Committee of Jessenius Faculty of Medicine in Martin (Approval no. EK39/2018). All the patients were diagnosed according to the ERS/ATS/WASOG consensus statement [13].

### 2.2. Exclusion Criteria

Patients with known history of cough-relevant comorbidities (gastroesophageal reflux disease, allergic rhinitis, bronchial asthma, acute or chronic upper or lower respiratory tract infections, state after pulmonary embolism, chronic heart failure, and ACE-I (ACE inhibitors) medication, chronic obstructive pulmonary disease, respiratory tract tumours, and other chronic diseases of airways or lung parenchyma) were excluded from the study.

### 2.3. Evaluated Parameters

Following parameters have been evaluated for the purpose of this study: age at diagnosis, gender, presence of signs and symptoms (fever, erythema nodosum, arthralgia, dyspnoea, cough, and chest pain), radiographic staging of sarcoidosis, results of analysis of BALF cellular component via flow cytometry (proportion of neutrophils, eosinophils, and lymphocytes in % and proportion of CD4+ to CD8+ T-lymphocytes), lung function (forced vital capacity (FVC), forced expiratory volume in 1 second (FEV1), maximal expiratory flows at 25 and 50% of FVC (MEF25 and MEF50), lung diffusion capacity for carbon monoxide (DLCO)), serum concentration of calcium, and the amount of calcium excreted in the urine per 24 hours.

#### 2.3.1. Chest X-Ray

Evaluation of the radiographic disease stage was performed using plain chest X-ray radiograph in PA projection in accordance with international guidelines [13]. The classic Scadding staging score was used: stage 0—no disease presentation on native chest X-ray; stage I—enlargement of hilar lymph nodes, without visible presentation in lung parenchyma; stage II—simultaneous presence of hilar lymph node enlargement and lung parenchyma involvement; stage III—visible presence of lung parenchyma affection without hilar lymph node enlargement; stage IV—pulmonary fibrosis (reticular pattern, hilar apicalization, and hemithorax retraction).

#### 2.3.2. Lung Function Testing

Lung function testing was performed on daily-calibrated open circuit device (MasterLab Body, Erich Jaeger, Germany; software version 4.2). It was performed in accordance with international guidelines [14]. Accepted FVC and FEV1 values were taken from those of three consequent manoeuvres of forced exhalation fulfilling the acceptance criteria, in which the sum of FEV1 + FVC was highest, and the difference between two highest values had to be less than 10% or 150 ml.

Measurement of single-breath lung diffusion capacity for carbon monoxide (DLCOsb) was performed on daily-calibrated open circuit device (BodyScope Ganshorn with HeliRapid and PowerCube units (Germany, software version 8.4F)). The accepted DLCO value was obtained as a mean value from two measurements fulfilling the acceptance criteria, in which the difference of DLCOsb figure was less than 10% or 1 mmol/kPa/s.

All evaluated lung function data were recorded as the percentage of reference values. For spirometric data, the reference dataset of ECSC was used. For DLCO values, the reference dataset by Salorinne was applied [15].

#### 2.3.3. Analysis of Cellular Component of BALF

Bronchoalveolar lavage fluid (BALF) was obtained during the tracheobronchofibroscopic examination by using the flexible bronchoscope (Olympus BF TYPE 1T10 (Japan)), wedged usually in middle lobar bronchus orifice, by fractionated instillation of isotonic sterile saline solution heated to 37°C with pH 7.0–7.2 with total volume of 100–140 ml. The lavage was considered as acceptable if the recovery volume was over 50% of the instilled volume.

Flow cytometry of BALF was performed immediately after its sampling using the FACSort device (Becton Dickinson). The samples were prepared by filtration and centrifugation at 1400 rpm for 10 min, PBS buffering, labelling by monoclonal antibodies (Becton Dickinson), 15–30 min incubation by room temperature at dark, and addition of stabilising solution. Percentage of lymphocytes, neutrophils, eosinophils, and CD4+ to CD8+ (CD4/CD8) T-lymphocytes in the overall cellular population was recorded.

#### 2.3.4. Calcemia and Calciuria

Methods for the examination of calcium serum levels and the amount of calcium excreted in the urine per 24 hours are not listed, as these examinations are routinely performed by validated methodology. Given the intraindividual variability of calcemia and calciuria values, the accepted values were represented by an average of measurements from three independent samples obtained in three consecutive days. If three samples were not available, the only average of measurements of two samples or a single performed measurement was accepted.

### 2.4. Statistical Analysis

Data are given as means (SEM), medians (range), or as proportions stated as percentages where appropriate. For the evaluation of the statistical significance, the group comparisons using *χ*^2^ test, Mann–Whitney test, or unpaired *t*-test were used as appropriate in GraphPad Prism 7.0 software. *p* < 0.05 was considered as statistically significant.

## 3. Results

### 3.1. Demographic and Clinical Characteristics of the Cohort

Sarcoidosis patients (*n* = 101), 56 women, with an average age at the time of diagnosis 42 ± 1.1 years (25–70 years) were included in the study. Sixty-five patients indicated the presence of cough, and 36 patients did not indicate this symptom. Distribution of patients by radiographic stage is summarised in Table 1. Most of the patients were in the second stage of the disease.

### 3.2. Relation of Cough to Smoking History

**Figure 1:**
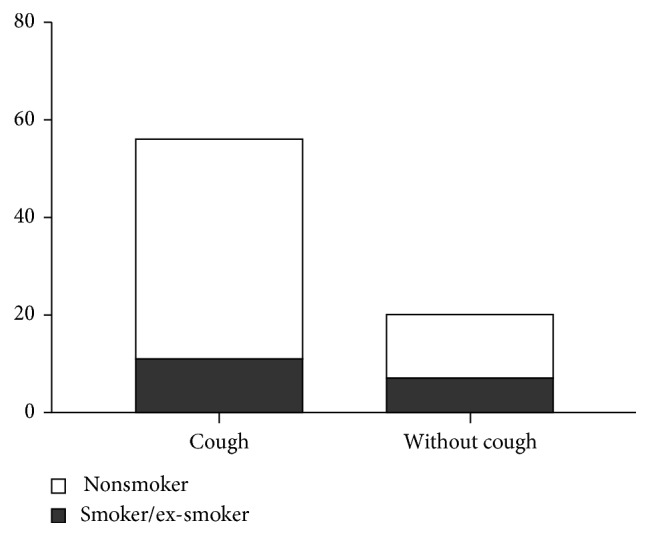
Cough in relation to the smoking history (*p*=0.1615).

Cough was present in 61.1% of smokers or ex-smokers and in 77.6% of nonsmokers (Figure 1). Cough was more frequent in nonsmokers although this difference was not statistically significant (*p*=0.16554).

### 3.3. Relationship of Cough to the Stage of Disease

**Figure 2:**
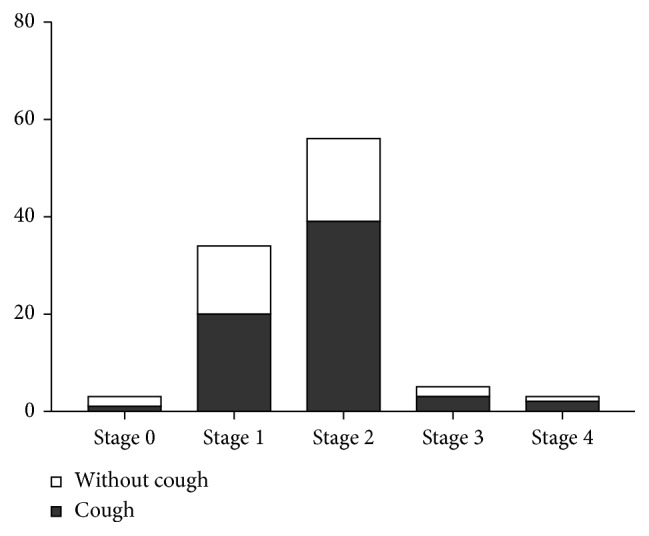
Cough in relation to the stage of disease (*p*=0.6548).

In our study, there were no significant differences in cough presence among sarcoidosis patients in relation to the stage of the disease. Cough was present in 33.3% of patients in stage 0, in 58.8% of patients in stage 1, in 69.6% of patients in stage 2, in 60% of patients in stage 3, and in 66.6% of patients in stage 4 (Figure 2).

### 3.4. Cough and Other Sarcoidosis Symptoms

**Figure 3:**
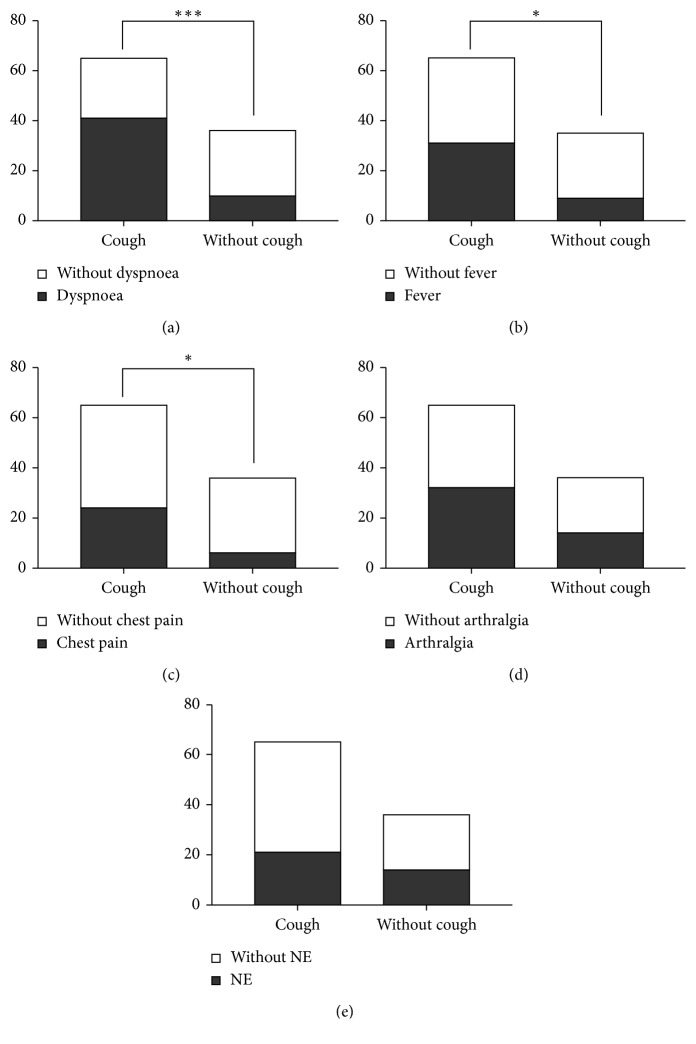
Cough in relation to other sarcoidosis symptoms: dyspnoea (^*∗∗∗*^*p*=0.0007) (a), fever (^*∗*^*p*=0.0324) (b), chest pain (^*∗*^*p*=0.0206) (c), arthralgia (^*∗*^*p*=0.3715) (d), and erythema nodosum (NE) (^*∗*^*p*=0.5056) (e).

Furthermore, we analysed the association of cough to other sarcoidosis symptoms: dyspnoea, fever, chest pain, erythema nodosum, and arthralgia. We found that cough was significantly associated with dyspnoea (*p*=0.0007). Dyspnoea was present in 63.1% of sarcoidosis patients with cough, while only 27.8% of sarcoidosis patients without cough reported this symptom (Figure 3(a)). Cough was also significantly associated with fever (*p*=0.0324), which was present in 47.7% of sarcoidosis patients with cough and only in 25.7% of sarcoidosis patients without cough (Figure 3(b)). Cough was also significantly associated to chest pain (*p*=0.0206), which was significantly more frequently reported by 36.9% of sarcoidosis patients with cough, against only in 16.7% of sarcoidosis patients without cough (Figure 3(c)). We did not find any significant association between cough and arthralgia manifestation (*p*=0.317) or cough and presence of erythema nodosum (*p*=0.505) in sarcoidosis patients, as shown in Figures 3(d) and 3(e).

### 3.5. Relationship of Cough to Endoscopic Findings

**Figure 4:**
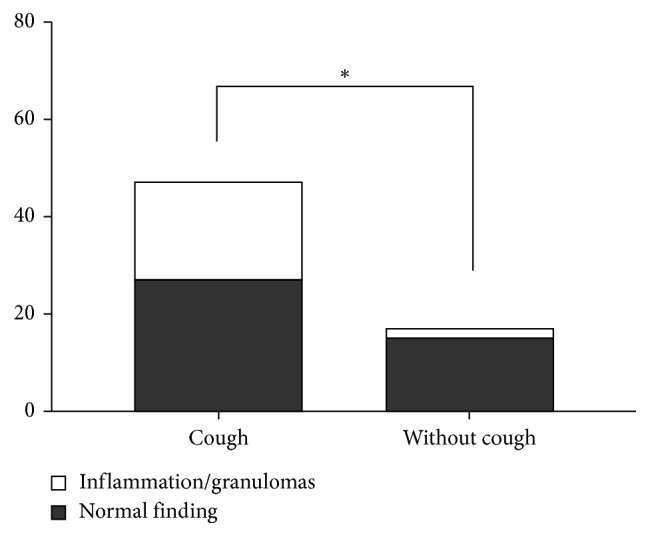
Cough in relationship to endoscopic finding (macroscopic changes of the tracheobronchial tree: inflammation or granulomas) (^*∗*^*p*=0.0206).

**Figure 5:**
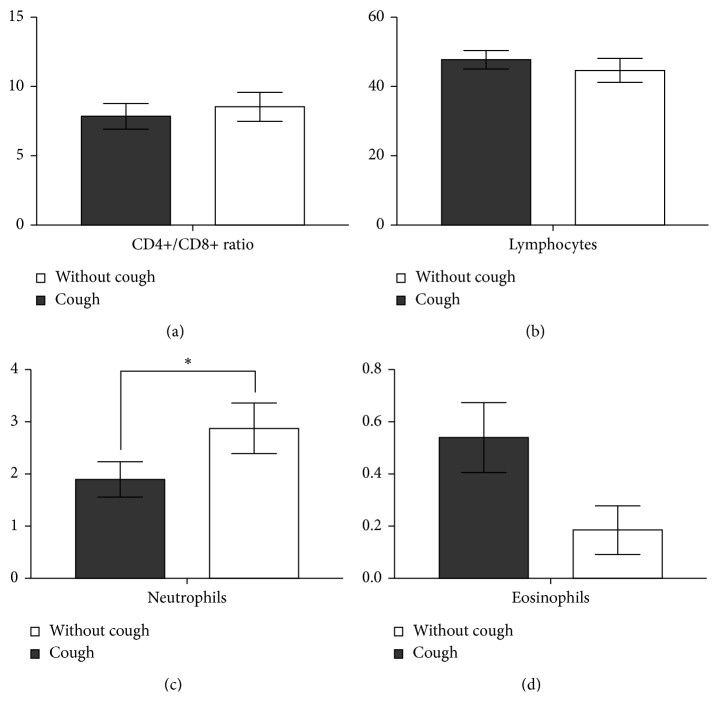
Cough in relation to results of flow cytometry of BALF: the relationship of cough to CD4+/CD8+ T-cell ratio (*p*=0.2614) (a), the proportion of lymphocytes (*p*=0.4289) (b), neutrophils (^*∗*^*p*=0.0252) (c), and eosinophils (*p*=0.1236) (d) in BALF.

Sarcoidosis patients reporting cough had significantly more often positive endoscopic finding, 42.6%, compared to sarcoidosis patients without cough, 11.8% (Figure 4). We did not find a significant difference in CD4+/CD8+ T-cell ratio in BALF (Figure 5(a)). Sarcoidosis patients with cough had significantly (*p*=0.0252) lower proportion of neutrophils in BALF (Figure 5(c)) compared with sarcoidosis patients without cough. The proportion of lymphocytes and eosinophils in BALF did not differ significantly (Figures 5(b) and 5(d)).

### 3.6. Relationship of Cough to Lung Function Testing

**Figure 6:**
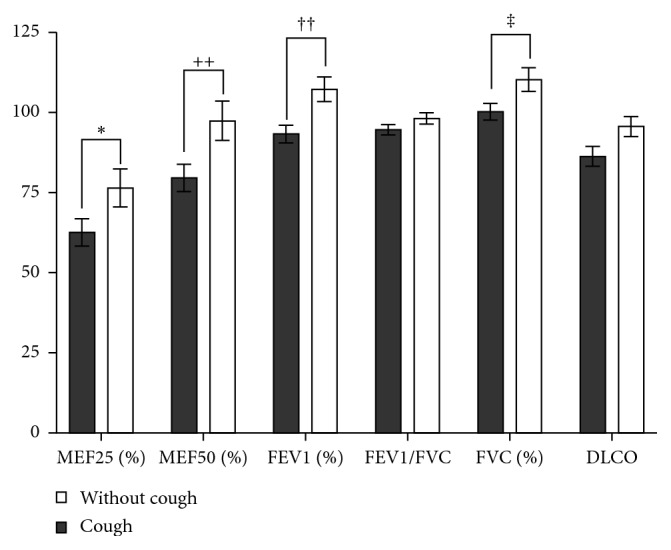
Cough in relation to the results of lung function testing. MEF25 (%)—maximal expiratory flow at 25% of forced vital capacity (expressed as % of the predicted value), MEF50 (%)—maximal expiratory flow at 50% of forced vital capacity (expressed as % of the predicted value), FEV1 (%)—forced expiratory volume in the first second (expressed as % of the predicted value), FVC—forced vital capacity (expressed as % of the predicted value), FEV1/FVC—ratio of FEV1 and FVC, DLCO—lung diffusion capacity for carbon monoxide (^*∗∗*^*p*=0.0304; ^++^*p*=0.0061; *p*=0.0025; *p*=0.0257).

In our study, we found significant differences in spirometric parameters between sarcoidosis patients with presence or absence of cough. Sarcoidosis patients with cough had significantly lower values of MEF25% predicted (maximal expiratory flow at 25% of the forced vital capacity) (*p*=0.0304) and MEF50% predicted (maximal expiratory flow at 50% of the forced vital capacity) (*p*=0.0061). These parameters are considered to indicate obstruction of small airways and may be used as a surrogate of early small airways disease. Values of forced expiratory volume in 1 s (FEV1)% predicted and FVC% predicted were also significantly lower in sarcoidosis patients with cough compared to those of sarcoidosis patients without cough (*p*=0.0025 and *p*=0.0257, respectively). Values of FEV1/FVC and DLCO did not differ significantly in these two groups of patients (Figure 6).

### 3.7. Relationship of Cough and Calcium Metabolism: The Potential Role of Vitamin D

**Figure 7:**
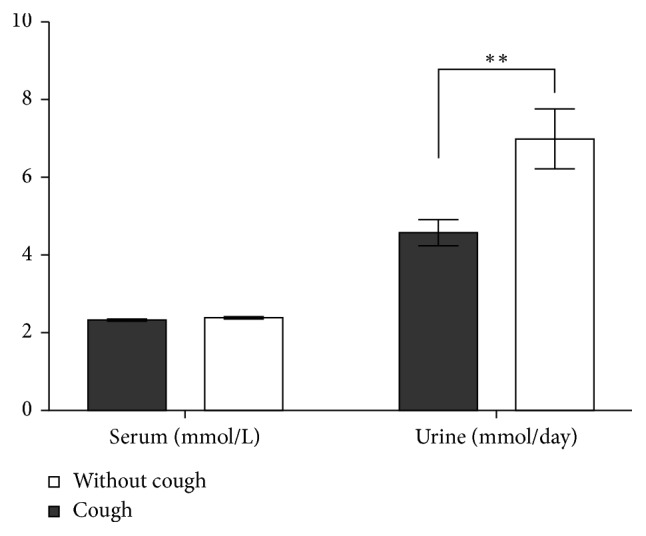
Cough in relationship to calcium level in serum and in urine (^*∗∗*^*p*=0.0014).

Sarcoidosis patients without cough had a significantly higher average value of calciuria compared to sarcoidosis patients with cough (6.988 ± 0.77 vs. 4.576 ± 0.337 mmol/day) (*p*=0.0014). Average values of calcaemia did not differ significantly in sarcoidosis patients without cough versus sarcoidosis patients with cough (2.332 ± 0.025 vs. 2.391 ± 0.0296 mmol/l) (Figure 7).

## 4. Discussion

Cough is a common and troublesome symptom of sarcoidosis, with a negative impact on quality of life. In the study of Sinha et al. [7], where cough frequency was measured by objective 24-hour cough monitor, approximately half of patients with sarcoidosis coughed more frequently than healthy controls. In this study, few patients with increased cough frequency did not report chronic cough. So some discordances exist between objective and subjective measures of cough [16]. However, subjective assessment of cough is perhaps more important than objective cough measurements, as it represents patients' perception of illness better. In our study, we performed a retrospective analysis of information that was available in patients' history and medical records. We analysed the association of presence or absence of cough and other sarcoidosis symptoms, as it was subjectively reported by sarcoidosis patients.

Epidemiological data from specialized cough clinics indicate that subjects reporting chronic cough are predominantly women [17]. It is known that there are gender differences in the maturation and physiology of the cough reflex, which can contribute to this gender-specific finding [18]. When we analysed the relationship of cough to gender, we found that cough was present slightly more often in female patients, although the difference was not significant. Our results are in agreement with the study of Harrison [8], who also found that cough is more prevalent in female sarcoidosis patients compared to male patients. Moreover, women have been found to have higher Scadding stages and more frequent pulmonary symptoms than men in sarcoidosis cohorts [9, 19].

Tobacco smoking is a well-known strong risk factor for several pulmonary diseases such as lung cancer or COPD. On the contrary, several studies showed that smokers have a lower risk of sarcoidosis [9, 20–22]. Similarly, our study showed that most of the sarcoidosis patients were nonsmokers. Furthermore, we found that cough was more prevalent in nonsmokers with sarcoidosis, although the difference was not statistically significant. Recent studies have shown that in otherwise healthy smokers, cough reflex sensitivity is diminished relative to that of nonsmokers. On the contrary, smoking cessation leads to prompt enhancement of cough reflex sensitivity, even after many years of smoking. One proposed mechanism is chronic cigarette smoke-induced desensitization of airway cough receptors [23].

In the study of Judson et al. [19], there was a trend for the cough to be worse in patients with higher Scadding stage. The associations between cough measures they examined and Scadding stage were not strong. We did not find significant differences in cough occurrence in sarcoidosis patients with various stages of the disease in our study; however, there are certain tendencies, which should be discussed.

A more common occurrence of cough in patients manifesting with dyspnoea and chest pain suggests a possible relation to the intrathoracic manifestation of disease activity. It can be assumed that the presence of granulomatous inflammation of lower respiratory tract is one of the key moments of either development or facilitation of cough in these patients what is suggested by documented significantly more common occurrence of cough in patients with macroscopic inflammatory changes of tracheobronchial tree in fibroendoscopy in our cohort. On the contrary, presented the association with increased body temperature could suggest a relation to the systemic inflammatory response. It should be assumed that systemic inflammatory response correlates with the intensity of inflammation of the tracheobronchial tree. Neither of the mentioned assumptions can be backed up by evidence due to retrospective characteristics of the study.

We also show lower (but not low—there is no lower limit) proportion of neutrophils in BALF in patients with cough. No direct pathogenetic explanation is being offered; however, it can be a bias—increased proportion of neutrophils caused by tobacco smoking. On the contrary, in our data, cough occurred in smokers less frequently than in nonsmokers.

Lung function testing plays an important role in the management and treatment monitoring of sarcoidosis patients. FVC has been recommended by an international group of sarcoidosis experts as the favoured objective endpoint indicating the severity of the disease [24]. In addition, clinicians tend to focus on the objective test, including lung function measurements, to assess the efficacy of antisarcoidosis therapy. However, significant discrepancy between radiographic severity of the disease and lung function impairment is often seen in sarcoidosis. By some authors, cough in sarcoidosis patients is independent of spirometric results [7, 19]. In our study, we found that patients with cough had significantly lower values of MEF25, MEF50, FEV1, and FVC in % of predicted values. Values of FEV1/FVC and DLCO did not differ significantly between these two groups of patients.

In our study, sarcoidosis patients without cough had a significantly higher average value of calciuria, compared to sarcoidosis patients with cough. Unfortunately, levels of vitamin D were not routinely examined in our cohort of sarcoidosis patients. But if we expect that patients with hypercalciuria have higher levels of calcitriol, we can speculate that increased calcitriol could have a protective effect on cough. Further research is needed to elucidate this question.

Management of cough in sarcoidosis still represents a clinical problem. Although our data might suggest an anti-inflammatory treatment (ideally locally applied to lower respiratory tract mucosa) as pathogenetically sound, the current guidelines [25] do not recommend the use of inhalation corticosteroids in this indication based on result analysis of randomized controlled studies. Mentioned guidelines recommend neuromodulator gabapentin, opioids, and speech pathology management for cough treatment in interstitial pulmonary diseases. These modalities are not a part of standardized treatment in sarcoidosis [26]. Our finding on the lower occurrence of cough in hypercalciuria allows speculation about the possible protective influence of calcitriol. Its possible therapeutic use should be considered a contraindication for risks arising from hypercalcemia and hypercalciuria.

The main limitation of our study is the analysis of retrospective data, and despite careful analysis of patient's history, we are not able to guarantee that some patients included in our cohort could have a coincidence of sarcoidosis and other unknown cough-relevant comorbidities.

Objective exclusion of cough-relevant comorbidities is quite difficult even in prospective studies because it requires implementation of many auxiliary examinations, which are often invasive (e.g., oesophageal pH monitoring and manometry to exclude GERD).

## 5. Conclusion

We found significant differences between sarcoidosis patients with and without the presence of cough regarding symptoms, spirometry, endobronchial findings, and calcium metabolism. Further research is needed to understand the etiopathogenetic mechanisms of cough in sarcoidosis patients.

## Figures and Tables

**Table 1:** Distribution of sarcoidosis patients by stage.

Scadding stage	Number of patients	Proportion (%)
0	3	2.97
1	34	33.66
2	56	55.45
3	5	4.95
4	3	2.97

The radiographic stage was determined by the evaluation of native chest X-ray (in accordance with the current valid international recommendations [13]).

## Data Availability

The data used to support the findings of this study are available from the corresponding author upon request.
